# A new herrerasaurid (Dinosauria, Saurischia) from the Upper Triassic Ischigualasto Formation of northwestern Argentina

**DOI:** 10.3897/zookeys.63.550

**Published:** 2010-10-19

**Authors:** Oscar A. Alcober, Ricardo N. Martinez

**Affiliations:** Museo de Ciencias Naturales, San Juan 5400, Argentina

**Keywords:** Dinosauria, Saurischia, Herrerasauridae, Carnian, Ischigualasto

## Abstract

Herrerasauridae comprises a basal clade of dinosaurs best known from the Upper Triassic of Argentina and Brazil, which have yielded remains of Herrerasaurus ischigualastensis and Staurikosaurus pricei, respectively. Systematic opinion regarding the position of Herrerasauridae at the base of Dinosauria has varied. Here we describe a new herrerasaurid, Sanjuansaurus gordilloi **gen. n.**, **sp. n.**, based on a partial skeleton from Carnian-age strata of the the Upper Triassic Ischigualasto Formation of northwestern Argentina. The new taxon is diagnosed by numerous features, including long, band-shaped and posterolaterally oriented transverse process on the posterior cervical vertebrae; neural spines of the sixth to eighth dorsal vertebrae, at least, bearing acute anterior and posterior processes; scapula and coracoid with everted lateral margins of the glenoid; and short pubis (63% of the femoral length). Phylogenetic analysis placed Sanjuansaurus within a monophyletic Herrerasauridae, at the base of Theropoda and including Herrerasaurus and Staurikosaurus. The presence of Sanjuansaurus at the base of the Ischigualasto Formation, along with other dinosaurs such as Herrerasaurus, Eoraptor, Panphagia, and Chromogisaurus suggests that saurischian dinosaurs in southwestern Pangea were already widely diversified by the late Carnian rather than increasing in diversity across the Carnian-Norian boundary.

## Introduction

Herrerasauridae (Benedetto 1973) is a clade of basal saurischian dinosaurs best known from the Upper Triassic of Argentina and Brazil (Reig 1963; Colbert 1970; Sereno and Novas 1992). Their phylogenetic position has varied in recent analyses from a position as sister-group of Dinosauria (Gauthier 1986; Brinkman and Sues 1987; Benton 1990; Novas 1992; Fraser et al. 2002), basal theropods (Sereno and Novas 1992; Sereno 1994, 1999; Sereno et al. 1993; Novas 1994, 1996, 1997; Rauhut 2003; Ezcurra and Cuny 2007; Ezcurra and Novas 2007; Bittencourt and Kellner 2009), or sister-group of Theropoda + Sauropodomorpha (Padian and May 1993; Bonaparte and Pumares 1995; Holtz 1995; Langer et al. 1999; Galton 2000; Langer 2004; Benton 2006; Ezcurra 2006; Irmis et al. 2007; Smith et al. 2007; Martinez and Alcober 2009). Resolving the phylogenetic position of Herrerasauridae is hindered by incomplete specimens and differences in character selection and scoring between analyses.

The close relationship between its two best known genera, Herrerasaurus and Staurikosaurus is well supported by a suite of synapomorphies (Novas 1994, 1996, 1997; Sereno and Novas 1994; Sereno 1999; Kellner and Campos 2000; Rauhut 2003; Langer 2004; Langer and Benton 2006; Bittencourt and Kellner 2009). Chindesaurus bryansmalli from the Norian Chinle Formation of the southwestern United States (Long and Murry 1995) has also been identified as a herrerasaurid (Long and Murry 1995; Novas 1997; Sereno 1999; Nesbitt et al. 2009) or as a more basal taxon (Langer 2004; Nesbitt et al. 2007; Bittencourt and Kellner 2009).

Here we describe a new herrerasaurid from strata near the basal contact of the Ischigualasto Formation. The new taxon is based on an associated, partially articulated skeleton recovered from Ischigualasto Provincial Park in 1994.

### Geological and palaeontological settings

**Figure 1.**
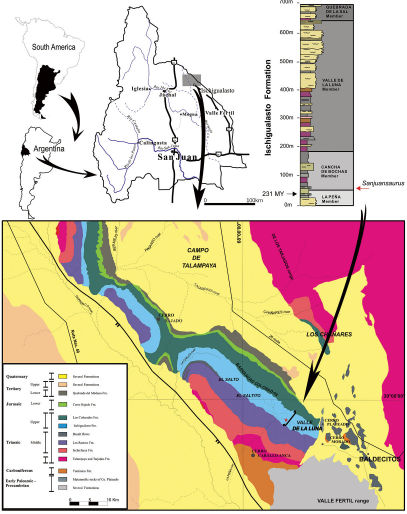
Geological map of the Ischigualasto – Villa Unión Basin (northwestern Argentina) and section of the Ischigualasto Formation at the type locality. The red star indicates the site of the holotype of Sanjuansaurus gordilloi (PVSJ 605), near the base of the Ischigualasto Formation.

The holotype of the new taxon (PVSJ 605) was found in 1994 during fieldwork carried out by the Instituto y Museo de Ciencias Naturales of the Universidad Nacional de San Juan. The Ischigualasto Formation crops out in northwestern Argentina and forms part of the Ischigualasto-Villa Unión Basin (Figure 1). It reaches up to 700 m in thickness and comprises fluvial channel sandstones with sandstones and mudstones deposited on a well-drained floodplain. Interlayered volcanic ashes 20 m above the base of the formation provide chronostratigraphic control and have yielded an age of 231.4 Ma, placing them in the Carnian stage (Rogers et al. 1993; Renne et al. 2010).

The Ischigualasto Formation is divided into four members (Currie et al. 2009). From the base to the top they are: the La Peña (from the base to 40 m), the Cancha de Bochas (40 to 180 m), the Valle de la Luna (180 to 650 m) and the Quebrada de la Sal (650 to 700 m) members (Figure 1). The La Peña Member consists of multi-story channel sandstones and conglomerates covered by poorly-drained floodplain mudstones. The Cancha de Bochas Member is composed of thick, well-drained floodplain mudstones interbedded with high-sinuosity channel sandstones. The Valle de la Luna Member is mostly characterized by amalgamated high-sinuosity channels, abandoned channels and marsh deposits. Finally, the Quebrada de la Sal Member consists of tabular fluvial deposits.

The new fossil was excavated at the La Gallinita locality, which is located in the lowest levels of the Cancha de Bochas Member. Itwas found 40 m above the base of the formation. Dinosaurs, including several specimens of Herrerasaurus ischigualastensis and Eoraptor lunensis, the holotype of Panphagia protos, and other as yet undescribed species (Martinez et al. 2008), carnivorous and herbivorous cynodonts, rhynchosaurs, and crurotarsan archosaurs were recovered from the same level.

## Methods

### Preservation and preparation

The reddish brown bones were covered by a coarse hematite crust, and the entire specimen was embedded in a grey-green, fine-grained sandstone matrix. The overall preservation of the specimen is good. All the bones are three-dimensionally preserved, and most are complete with the exception of the femora, which are partially distorted and lack fine details. The incompleteness of the skeleton is attributable to pre-burial processes, although the third, fourth and anterior part of the fifth dorsal vertebra were lost in the course of preparation. The specimen was prepared using a pneumatic air scribe and pin vice.

### Terminology

We employ traditional, or “Romerian,” anatomical and directional terms rather than their veterinarian alternatives (Wilson 2006). “Anterior” and “posterior”, for example, are used as directional terms rather than “rostral” or “cranial” and “caudal”. We also follow Wilson’s (1999) recommendations regarding the identification of vertebral laminae in saurischians.

We used the stem-based phylogenetic definition for Herrerasauridae proposed by Sereno (Sereno 2005) rather than the node-based definition (Sereno and Novas 1992, Langer 2004), obviating the need for a suprafamilial taxon (Herrerasauria; Langer 2004). We thus define Herrerasauridae as "the most inclusive clade containing Herrerasaurus ischigualastensis but not Passer domesticus" (Sereno 2005).

### Phylogenetic Analysis

In order to asses the phylogenetic position of the new taxonamong basal Dinosauria, we added it (Table 1) and the recently described basal sauropodomorph Panphagia (Martinez and Alcober 2009) to the character-taxon matrix published by Langer and Benton (2006). We also modified several character states for these basal taxa following Martínez and Alcober (2009). The software used to analyze the phylogenetic relationships was TNT 1.1 (Goloboff et al. 2003).

**Table 1.** Character state scores for Sanjuansaurus gordilloi (PVSJ 605). Data lines inserted into the data matrix of Langer and Benton [33] with the addition of Martinez and Alcober [30]

Sanjuansaurus	0???? ?00?? ????? ????? ??000 00?11 11111 1?110 00111 1???? ????? ????? ??1?? ???2? ?1??0 010?? ?0001 00000 0000? ???

### Nomenclatural Acts

This published work and the nomenclatural acts it contains have been registered in ZooBank, the proposed online registration system for the ICZN. The ZooBank LSIDs (Life Science Identifiers) can be resolved and the associated information viewed through any standard web browser by appending the LSID to the prefix “http://zoobank.org/”. The LSID for this publication is: urn:lsid:zoobank.org:pub:FB2AE660-C3EE-4348-BF9F-F4311C47E853

### Institutional abbreviations:

PVSJInstituto y Museo de Ciencias Naturales, San Juan 5400, Argentina.

## Results

### Systematic Paleontology

**Systematic hierarchy**

**Dinosauria Owen, 1842**

**Saurischia Seeley, 1887**

**Herrerasauridae Benedetto, 1973**

#### Sanjuansaurus gen. n.

urn:lsid:zoobank.org:act:DC75ADA0-0C6B-41D5-8E29-CD76725FD704

##### Etymology:

*Sanjuan*, in reference to San Juan Province, Argentina; *saurus*, lizard (Latin).

##### Type species:

Sanjuansaurus gordilloi

##### Sanjuansaurus gordilloi sp. n.

urn:lsid:zoobank.org:act:84F081D6-4E0A-414E-83A3-D994263C1005

###### Etymology:

*gordilloi*, in honor of Raul Gordillo, head fossil preparator and artist in the laboratory of the San Juan Museum and team member during many years of excavation.

###### Holotype:

PVSJ 605, an incomplete skeleton including left maxilla, partial axial column, from the axis to the twelfth caudal vertebra, lacking the third, fourth, and anterior half of the fifth dorsal vertebrae, both scapulae, left ulna, ungual phalanx of left? digit III, preacetabular portion of the left ilium, proximal end of left and complete right pubis, both femora and tibiae, right fibula, right astragalus and calcaneum, and left metatarsal II.

###### Type locality:

The specimen was found in the Cancha de Bochas Member (Currie et al. 2009), 40 m above the base of Ischigualasto Formation. The type locality, informally called “Herrera de la base”, is located 3 km northwestern of “Cancha de Bochas” locality, Ischigualasto Provincial Park, San Juan, Argentina (Figure 1).

###### Horizon and age:

40 m above the base of the Ischigualasto Formation, Late Triassic, Carnian (ca. 231.4 Ma), Ischigualasto–Villa Unión Basin (Rogers et al. 1993; Renne et al. 2010). The type horizon lies at approximately the same level as the dated ash, which implies a late Carnian age for the specimen.

###### Diagnosis:

Diagnosed bythe followingautapomorphies: shelf-like, posterolaterally directed transverse processes on the posterior cervical vertebrae; neural spines of the sixth to eighth dorsal vertebrae, at least, bearing acute anterior and posterior processes; everted lateral margins of the glenoid; short pubis (63% of the femoral length); and pronounced, rugose scar on the medial surface of the femur at the level of the fourth trochanter.

These features distinguish Sanjuansaurus gordilloi from the previously described herrerasaurids, such as Herrerasaurus ischigualastensis and Staurikosaurus pricei, as well as other basal saurischians from Ischigualasto Formation, such as Eoraptor lunensis (Sereno et al. 1993), Panphagia protos (Martinez and Alcober 2009), and Chromogisaurus novasi (Ezcurra 2008, 2010)

## Description

**Figure 2.**
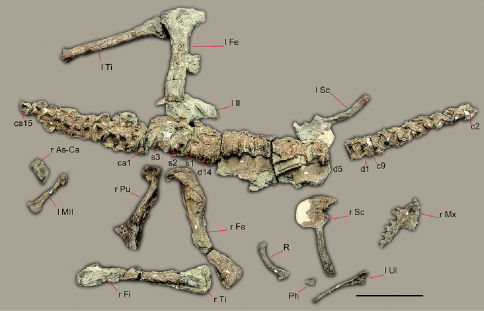
Preserved bones of Sanjuansaurus gordilloi (PVSJ 605), positioned as they were found in the field. *Abbreviations*: **ca1–15**, caudal vertebrae 1–15; **c2–9**, cervical vertebrae 2–9; **d1–14**, dorsal vertebrae 1–14; **l Fe**, left femur; **l il**, left ilium; **l MII**, left metatarsal II; **l Sc**, left scapula and coracoid; **l Ti**, left tibia; **l Ul**, left ulna; **Ph**, manual phalanx; **R**, rib; **r As-Ca**, right astragalus and calcaneum; **r Fe**, right femur; **r Fi**, right fibula; **r Fe**, right femur; **r Mx**, right maxilla; **r Pu**, right pubis; **r Sc**, right scapula and coracoid; **r Ti**, right tibia; **s1–3**, sacral vertebrae 1–3. Scale bars equals 20 cm.

**Figure 3.**
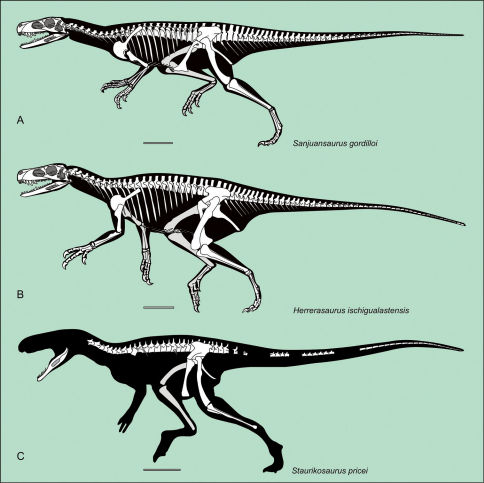
Silhouette reconstruction of the skeletons of the best known herrerasaurids.Sanjuansaurus gordilloi (PVSJ 605) (**A**) Herrerasaurus ischigualastensis (**B**) Staurikosaurus pricei (**C**) Scale bars equals 20 cm. **A**: missing bones of Sanjuansaurus modified from Sereno 1994; B: from Sereno 1994; C: from Novas 1997.

Although some bones of Sanjuansaurus gordilloi were disarticulated, the proximity of all elements, found in an area of only one square meter, their complementary size, and the absence of any duplicated elements suggest that they represent a single individual (Figure 2). In size and general proportions (Table 2, 3), the new specimen (Figure 3A) is comparable to a medium-sized Herrerasaurus (Figure 3B), and slightly larger than the only known specimen of Staurikosaurus (Figure 3C).

### Cranium.

**Figure 4.**
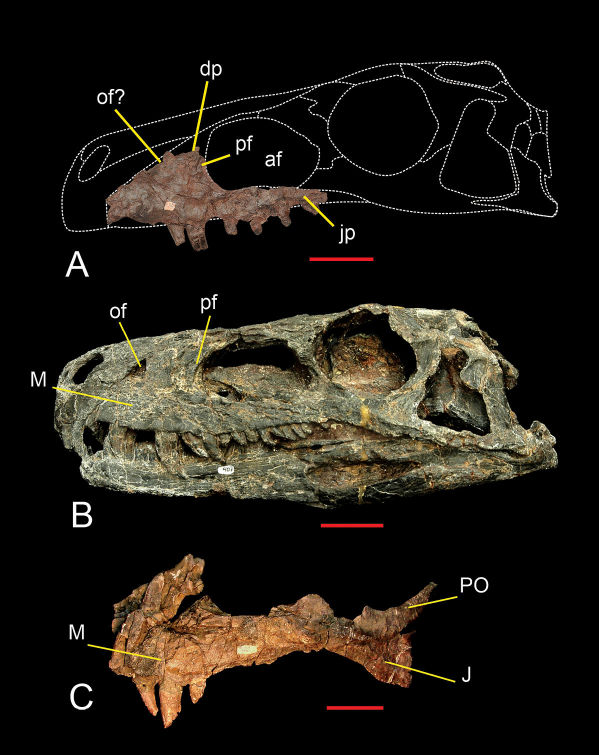
Maxilla of Sanjuansaurus gordilloi (PVSJ 605). Left maxilla of Sanjuansaurus in lateral view (**A**) Skull of Herrerasaurus (PVSJ 407) in left lateral view (**B**) Left maxilla of Herrerasaurus (PVSJ 053) in lateral view (**C**) *Abbreviations*: **af**, antorbital fossa; **dp**, dorsal process; **ip**, internarial process; **J**, jugal; **jp**, jugal process; **M**, maxilla; **of**, oval fenestra; **pf**, promaxillary fenestra; **PO**, postorbital. Scale bars equals 5 cm.

The left maxilla is the only cranial bone preserved (Figure 4A). It exhibits the anteroposteriorly elongated dorsal process and transversely narrow antorbital fossa as in Herrerasaurus (Figure 4B, C) but unlike the wide fossa of Eoraptor (Sereno et al. 1993: Fig. 2A). The anterodorsal border of the antorbital fossa shows a slit-shaped promaxillary fenestra as in Herrerasaurus, some coelophysoids*, Zupaysaurus*, and most tetanurans (Welles 1984; Witmer 1997; Arcucci and Coria 2003; Sereno 2007). The anterior border of the maxilla is slightly convex and preserves the posterior border of the subnarial foramen as in other saurischians. Breakage of the dorsal portion makes it impossible to determine whether there is an oval fenestra between the premaxilla and maxilla as in Herrerasaurus (Sereno and Novas 1994). The dorsal border of the jugal process below the antorbital fenestra is horizontal (Figure 4). In specimens of Herrerasaurus, this suture is either horizontal (PVSJ 053, holotype of “Frenguellisaurus ischigualastensis”) or posteroventrally inclined (PVSJ 407) (Sereno and Novas 1994: Fig. 1).

**Table 2.** Dimensions (mm) of preserved vertebrae (Figures 2, 3, 5) from the holotypic specimen of Sanjuansaurus gordilloi (PVSJ 605).

Vertebra	Centrum_length1	Vertebra	Centrum_length1
C2	37.0	D13	36.0
C3	42.5	D14	34.5
C4	44.0	S1	38.0
C5	46.1	S2	48.0
C6	47.5	S3	46.2
C7	45.6	CA1	35.8
C8	38.5	CA2	36.8
C9	38.0	CA3	35.0
D1	33.6	CA4	37.0
D2	28.3	CA5	33.2
D6	36.8	CA6	32.3
D7	38.0	CA7	38.5
D8	38.8	CA8	38.5
D9	36.6	CA9	37.8
D10	37.7	CA10	37.8
D11	37.5	CA11	36.5
D12	38.0		

**Table 3.** Dimensions (mm) of girdle and limb bones (Figures 2, 3, 6-8) of the holotypic specimen of Sanjuansaurus gordilloi (PVSJ 605).

Bone	Measurement	Length
Scapulocoracoid	Coracoid, maximum height	63.0
	Posterior process (glenoid to tip of process)	131.0
	Scapular length	185.0
	Scapular blade, minimum width	18.5
	Scapular blade, distal width	26.8
	Height (glenoid to acromion)	89.5
Ulna	Maximum length	178.2
	Anteroposterior shaft diameter (mid-shaft)	10.1
Pubis	Length (acetabulum to foot)	260.0
	Iliac peduncle length	32.0
	Ischial peduncle length	29.0
	Pubic foot, maximum length	100.2
	Pubic foot, maximum width	21.5
Femur	Maximum length	395.0
	Maximun distal width	90.5
Tibia	Length	260.0
	Maximun proximal dimension	91.0
	Proximal width	41.5
	Distal transverse width	40.0
	Distal anteroposterior width	43.5
Astragalus-Calcaneum	Proximodistal depth	36.0
	Transverse width	83.0
	Medial anteroposterior width	37.5
Metatarsal II	Length	147.5
	Maximun proximal dimension	40.0
	Distal transverse width	32.0

*Abbreviations*: **C** cervical; **CA** caudal; **D** dorsal; **S** sacral. **1**Measured along ventral edge excluding anterior convexity of centrum when present.

### Axial skeleton.

**Figure 5.**
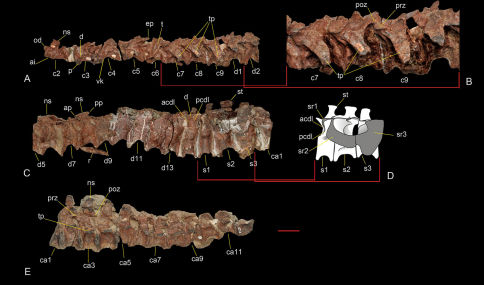
Axial skeleton of Sanjuansaurus gordilloi (PVSJ 605). Nine anterior cervical and two anterior dorsal vertebrae in left lateral view (**A**). Cervical vertebrae 7, 8 and 9 in left lateral view (**B**). Dorsal vertebra 1 to caudal vertebra 1 in left lateral view (**C**). Reconstruction of the sacrum in left lateral view (**D**). Caudal vertebra 1 to 12 in left lateral view (**E**). *Abbreviations*: **acdl**, anterior centrodiapophyseal lamina; **ai**, axis intercentrum; **ap**, anterior process of dorsal neural spine; **ca1–12**, caudal vertebrae 1 to 12; **c2–9**, cervical vertebrae 2 to 9; **d**, diapophysis; **d1–13**, dorsal vertebrae 1 to 13; **ep**, epipophysis; **ns**, neural spine; **od**, odontoides; **p**, parapophysis; **pcdl**, posterior centrodiapophyseal lamina; **prz**, prezygapophysis; **poz**, postzygapophysis; **pp**, posterior process of dorsal neural spine; **s1–3**, sacral vertebrae 1 to 3; **sr1–3**, sacral ribs 1 to 3; **st**, spine table; **t**, tooth; **tp**, transverse process; **vk**, ventral keel. Scale bar equals 5 cm.

The cervical vertebrae are preserved from the incomplete atlas to the last vertebra of the series (Figure 5A, B). Sanjuansaurus has nine cervical vertebrae. The last cervical vertebra differs from the first dorsal vertebra in the presence of a ventral keel and being nearly 30% longer, with the paraphophysis being more anteriorly located and the capitulum of the associated rib being more slender (Figure 2, 3, 5A). Although ten cervical vertebrae have been reported in Herrerasaurus (Sereno and Novas 1994), the cervicodorsal transition is not well preserved on any of the known specimens.

The atlas is represented only by its centrum, the odontoid (Figure 5A), which is completely co-ossified with the axial centrum. It is subcircular in dorsal view with a pointed median projection as in Herrerasaurus (Sereno and Novas 1994). It is semilunar in outline in anterior view. The dorsal and ventral surfaces are concave and convex, respectively, as in Herrerasaurus.

The axial intercentrum (Figure 5A) is fused to the axial centrum. It is much broader than the anterior end of the axial centrum as in Herrerasaurus. In lateral view it is subquadrangular rather than triangular as in Herrerasaurus (Sereno and Novas 1994). Its anteroposterior length is one-half that of the axis, resembling that in large specimens of Herrerasaurus (PVSJ 053), but proportionately longer than that in the small ones(e.g, PVSJ 407), where it equals one-third of axial centrum length. As in Herrerasaurus the axial intercentrum is deeply cupped anteriorly.

The axial centrum (Figure 5A) is twice as long as it is deep and bears a pronounced ventral keel as in Herrerasaurus (Sereno and Novas 1994). The diapophyses are short and face dorsolaterally as in Herrerasaurus, although they are located more anteriorly and are level with the axial intercentrum. In Herrerasaurus the diapophyses do not extend beyond the anterior articular surface of the centrum. Damage to the neural spine and the postzygapophyses preclude description of their structure.

The postaxial cervical centra are spool-shaped, amphicoelous, and have pronounced ventral keels. In Herrerasaurus, by contrast, the lateral and ventral sides of the centrum are less concave, and the ventral keel diminishes progressively in more distal vertebrae (Sereno and Novas 1994). The second to the sixth cervical centra are approximately parallelogram-shaped in lateral view whereas the seventh to the ninth centra are subrectangular. The third cervical vertebra is longer than the axis, and centrum length increases posteriorly up to the sixth cervical centrum (Figure 5A) as in Herrerasaurus (Sereno and Novas 1994). This condition is different from Staurikosaurus, in which the third or fourth centrum is the longest (Galton 1977; Bittencourt and Kellner 2009). The parapophyses of the anterior cervical vertebrae of Sanjuansaurus protrude ventrally beyond the ventral margin of the centrum in lateral view, in contrast to the condition in Herrerasaurus, where they are located slightly dorsal to the ventral border of the centrum. Posteriorly, from the seventh vertebra, the parapophyses are displaced progressively backwards and upwards. All the cervical neural spines are broken at their bases. From the third to the ninth vertebra, the prezygapophyses extend one third of the centrum length beyond the anterior border of the body. The postzygapophyses are high and do not project behind the level of the posterior face of the centrum. As in Herrerasaurus (Sereno and Novas 1994), the epipophyses are pointed and extend beyond the postzygapophyses (Figure 5A). On the sixth cervical vertebra, anterior and posterior centrodiapophyseal, postzygodiapophyseal, and prezygodiapophyseal laminae are present and become more prominent on successive cervical vertebrae. From the fifth cervical vertebra onwards, the transverse processes increase in both anteroposterior width and transverse length and project posteroventrolaterally (Figure 5B), an unusual shape among theropods. The transverse processes are triangular in Herrerasaurus (Sereno and Novas 1994) and Staurikosaurus (Galton 1977).

The dorsal vertebrae are articulated. Only the third, fourth and anterior half of the fifth vertebra are lacking and were accidentally lost during preparation (Figure 5A–C). Thus we can say with confidence that there are 14 dorsal vertebrae as in Herrerasaurus (Sereno 2007, *contra* Novas 1994), in contrast to 15 in Staurikosaurus (Galton 1977; Bittencourt and Kellner 2009). The dorsal vertebrae are characterized by anteroposteriorly short centra and tall neural arches as in Herrerasaurus and Staurikosaurus (Novas 1994; Colbert 1970; Bittencourt and Kellner 2009). They differ in having more distinctly spool-shaped centra (Figure 5C). The ventral concavity is very conspicuous in the first to eleventh dorsal vertebrae, decreasing slightly in the twelfth to fifteenth. Centrum length decreases from the first to the second dorsal, increases from the sixth to the twelfth, and decreases again to the final dorsal vertebra (Table 2). A ventral keel is absent on all dorsal vertebrae although a prominent keel is present on the last cervical vertebra. The parapophyses are prominent and oval in lateral view. On the first and second dorsals, they are located at mid-length of the centrum. In more posterior dorsal vertebrae, they are displaced anterodorsally. On the twelfth dorsal vertebra, the parapophyses and diapophyses are located at the same level. The parapophyses of the first and second dorsal vertebrae are shared between the centrum and neural arch. In the sixth vertebra (the first completely preserved vertebra posterior to the second), the parapophyses are located entirely on the neural arch (Figure 5B). The neural arches are anteroposteriorly short and dorsoventrally deep. Pre- and postzygapophyses are anteroposteriorly short, the former are slightly longer than the latter and extend beyond the anterior centrum face as in Herrerasaurus (Novas 1994). Pre- and postzygapophyses are separated by an interzygapohpyseal sulcus, which extends onto the anterior and posterior edges of the neural spine as in Herrerasaurus and Staurikosaurus (Novas 1994; Bittencourt and Kellner 2009). The second dorsal vertebra, which is disarticulated from the posterior part of the vertebral column, has a well developed hyposphene similar to that present in Herrerasaurus (Novas 1994). The remaining dorsals presumably also had hyposphene-hypantrum articulations, but these are obscured by the tight articulation between vertebrae. The anterior neural spines are broken off with the exception of those on the sixth and eighth dorsal vertebrae. These have distinctive pointed processes extending anteriorly and posteriorly from the apex of the spine (Figure 5C, D). The distal end of the neural spine of the last dorsal vertebrae also bears a spine table, which is similar to that in Herrerasaurus (Novas 1994). All the dorsal vertebrae have well developed anterior and posterior centrodiapophyseal, postzygodiapophyseal, and prezygodiapophyseal laminae. These laminae bound three subtriangular spaces the infraprezygapophyseal, infradiapophyseal and infrapostzygapophyseal fossae (Figure 5C). These fossae converge below a horizontal roof formed by the diapophysis and pre- and postzygodiapophyseal laminae as in Herrerasaurus.

The sacrum (Figure 5C, D) of Sanjuansaurus comprises three vertebrae. The first is a dorsosacral whereas the second and third represent the primordial sacral pair as in Herrerasaurus (Sereno 2007, *contra* Novas 1994). In Staurikosaurus the sacrum is composed of two primordial sacrals, with some uncertainty concerning the presence of a dorsosacral or caudosacral (Bittencourt and Kellner 2009). Given the degree of neurocentral coossification in other parts of the axial column and the fusion of the sacral ribs to their respective centra, it is surprising that the sacral centra are not co-ossified.

The first sacral vertebra is 10% longer than the last dorsal vertebra, and the position of the infradiapophyseal laminae and the transverse processes are somewhat different. The posterior centrodiapophyseal lamina is displaced anteriorly toward the anterior centrodiapophyseal lamina, so that ventral to the transverse process they both extend nearly vertically (Figure 5C, D). The transverse process is not a single flat process as on the posterior dorsal vertebrae but rather is composed of two laminae, one horizontal and the other one vertical, which join to form an inverted L-shape near the contact with the ilium. This configuration is similar to that shown on dorsal vertebra 15 in Herrerasaurus (Novas 1994: Fig. 1). The addition of the ventral lamina presumably strengthened the transverse process. The latter expands posterolaterally toward its distal end, which is broken away. The form of the process and its distal expansion suggest that it probably contacted the preacetabular process of the ilium, but this contact or the distal articular surface is not preserved. The distal end of the neural spine is expanded to form a spine table as in Herrerasaurus (Novas 1994). The anterior and posterior borders of the neural spine have median sulci that extend between the pre- and postzygapophyses, respectively.

The second sacral vertebra is 25% longer than the first and is the longest in the sacrum (Figure 5C, D). This is true for one individual of Herrerasaurus (PVSJ 373), whereas in another (PVL 2566) the third sacral vertebra is longest (Novas 1994). The centrum is transversally narrower and dorsoventrally flatter, and the neural spine is broader than in the first sacral vertebrae as in Herrerasaurus (Novas 1994). The spine table and anterior and posterior median sulci are twice the transverse width of the corresponding features on the first and third sacral vertebrae. The ribs are extensively fused to the anterodorsal portion of the centrum.

The third sacral vertebra, the most robust of the sacrum, is dorsolaterally flattened and transversally expanded as in Herrerasaurus (Novas 1994) (Figure 5C, D). As on the second sacral vertebra, robust sacral ribs are fused to the centrum. The ventral border of the rib is located at the same level as the ventral surface of the centrum, in contrast to the condition in Herrerasaurus, in which the rib is offset dorsally (Novas 1994). The neural spine is equal in height to the second sacral but is lateromedially narrower.

The sacral ribs of the second and third sacral vertebrae have broad distal attachment surfaces that are continuous and, in lateral view (Figure 5D) form a C-shape that opens dorsally. Large subcircular openings are present between the articular ends of the sacral ribs and the centra as in Herrerasaurus (Novas 1994).

The first 15 caudal vertebrae of PVSJ 605 are preservedin articulation. The posterior end of the fifteenth vertebra and all the hemal arches are lacking (Figure 5E). The preserved centra are strongly constricted, or spool-shaped, more so than in Herrerasaurus (Novas 1994: Fig. 4). As in Herrerasaurus and Staurikosaurus (Bittencourt and Kellner 2009), the centra lack ventral keels. The first caudal centrum is 10% shorter than that of the last sacral, and this length is maintained with minor variation along the preserved series, despite the decreasing height of the centra (Table 2). The neural arches are very tall with zygapophyses located far from the transverse process as in Herrerasaurus (Novas 1994). The subhorizontal transverse processes are situated in the middle of the centrum and project laterally and slightly posteriorly. In section the transverse processes are dorsoventrally flattened, in contrast to the semicircular section evident in Herrerasaurus (Novas 1994) and Staurikosaurus (Bittencourt and Kellner 2009). The only preserved neural spines, the third and fourth, are tall and near vertical, as is the case with the proximal caudal spines in Herrerasaurus. The caudal neural spines lack the anterior and posterior sulci present on the dorsal and sacral spines, and on the anterior caudals of Herrerasaurus (Novas 1994). The prezygapophyses extend beyond the anterior centrum face, whereas the postzygapophyses terminate flush with the posterior centrum face.

### Appendicular Skeleton.

**Figure 6.**
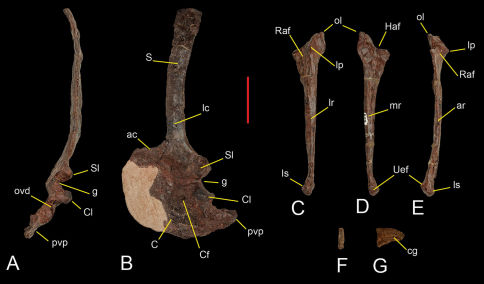
Preserved shoulder blade and forelimb bones of Sanjuansaurus gordilloi (PVSJ 605). Left scapula and coracoid inposterior(**A**), and lateral (**B**) views. Left ulna in lateral (**C**), medial (**D**), and anterior views (**E**). Ungual phalanx of digit III in proximal (**F**) and lateral view (**G**). *Abbreviations*: **ac**, acromion; **ar**, anterior ridge; **C**, coracoid; **cg**, collateral groove; **Cf**, coracoid foramen; **Cl**, coracoid lip; **g**, glenoid surface; **Haf**, humeral articular facet; **lc**, lateral crest; **lp**, lateral prominence; **lr**, lateral ridge; **ls**, ligament scars; **lp**, lateral prominence; **ol**, olecranon ; **ovd**, oval depression; **pvp**, posteroventral process; **Raf**, radius articular facet; **S**, scapula; **Sl**, scapular lip; **Uaf**, ulnare articular facet. Scale bar equals 5 cm.

Each scapula is firmly fused to its respective coracoid. The anterior margin is broken away on both scapulae, although its curved margin can be restored. The acromial process diverges from the blade at an angle slightly greater than 90°, as in Herrerasaurus (Sereno 1994). The glenoid is shared unevenly between the scapula and coracoid, the former contributing a smaller portion of the articulation, as in Herrerasaurus. The margin, or lip, of the glenoid protrudes laterally in a conspicuous manner, which does not seem to be an artifact of preservation (Figure 6A). The scapular blade is straplike with narrow proportions in lateral view (Figure 6A, B). The minimum width of the blade (near the base) is 54% the width of the acromial margin. This ratio is smaller than that found in either small (83%; PVSJ 407) or very large individuals (60%; PVSJ 053, “Frenguellisaurus”) of Herrerasaurus. The dorsal margin of the acromion is thin in contrast to the thickened border in Herrerasaurus (Sereno 1994). The lateral surface of the scapular blade has a distinct crest along the proximal two thirds of its length as in Herrerasaurus (Sereno 1994). In lateral view the blade is gently arched posteriorly in contrast to the nearly straight blade in Herrerasaurus.

The semicircular, plate-shaped coracoid (Figure 6A, B) is broader anteroposteriorly than dorsoventrally as in Herrerasaurus (Sereno 1994). It is gently concave medially. As in Herrerasaurus, the coracoid foramen is located anteroventral to the glenoid, opening anterolaterally entirely within the coracoid. The margin of the glenoid is particularly prominent laterally, forming a shelf (Figure 6A). The hook-shaped posteroventral process of the coracoid is long and pointed (Figure 6B), similar to that in ornithomimids such as Gallimimus (Osmólska and Barsbold 1990: Fig. 4). When the scapular blade is held vertically it is seen to extend far posterior to the glenoid. The process is considerably shorter in Herrerasaurus (Brinkman and Sues 1987: Fig. 2–10). The glenoid is separated from the posteroventral process by a notch marked by a deep depression (Figure 6A).

The ulna is more gracile than that of Herrerasaurus (Sereno 1994), but otherwise similar in shape. The proximal end bears a prominent olecranon process and a concave articular surface (Figure 6C–E). The lateral surface of the proximal end has a distinct protuberance that contributes to a concave articular surface for the proximal end of the radius as in Herrerasaurus (Sereno 1994). In Sanjuansaurus the ulnar protuberance is more acute. The medial surface of the proximal end is slightly concave, in contrast to the convex surface of Herrerasaurus, although this difference may be due to postmortem deformation in Sanjuansaurus. The distal half of the ulnar shaft shows a gentle medial curvature and has longitudinal ridges on its anterior, lateral and medial surfaces as in Herrerasaurus (Sereno 1994). The ulnar shaft of Sanjuansaurus appears to be more slender than in Herrerasaurus. The distal end of the ulna exhibits several differences to that of Herrerasaurus. It is expanded to a lesser degree than in small individuals of Herrerasaurus (e.g, PVSJ 373; Sereno 1994). The articular surface for the ulnare is concave and faces anteromedially (Figure 6D, E) in contrast to the convex surface of Herrerasaurus. Anterolateral to this articular facet, there is a protuberance that extends distally (Figure 6C–E). This protuberance may be homologous with a subtriangular ligament rugosity in Herrerasaurus (Sereno 1994).

Of the manus only one manual ungual (Figure 6F, G) is preserved. The ungual has been crushed transversely. Its curvature and transversely narrow proportions (Figure 6F) identify it as pertaining the the manus rather than the pes. Its small size, short and deep proportions, and ventral position of the attachment groove suggest that it is probably the fourth (terminal) phalanx of the third digit. The ungual has a well developed flexor tubercle more deeply grooved on the left side (Figure 6G). Although we considered that it is the smaller ungual of the hand, it is still small compared to individuals of Herrerasaurus (PVSJ 373) smaller in overall size than Sanjuansaurus.

**Figure 7.**
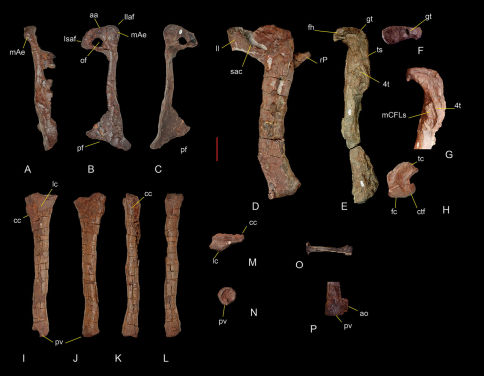
Pelvic and hind limb bones of Sanjuansaurus gordilloi (PVSJ 605). Right pubis in anterodorsal (**A**), lateral (**B**), and medial views (**C**). Left femur articulated with the preacetabular portion of the ilium and proximal end of the pubis in lateral view (**D**). Right femur in medial view (**E**). Right femur head in proximal view (**F**). Proximal end of the right femur in anteromedial view (**G**). Distal end of the left femur in distal view (**H**). Left tibia in lateral (**I**), medial (**J**), anterior (**K**), posterior (**L**), proximal (**M**), and distal views (**N**). Left metatarsal II in anterior view (**O**). Distal end of the right tibia in posterior view (**P**). *Abbreviations*: **aa**, acetabular area; **ao**, abnormal outgrowth in posteroventral process; **cc**, cnemial crest; **ctf**, crista tibiofibularis; **4t**, fourth trochanter; **fc**, fibular condyle; **fh**, femoral head; **gt**, greater trochanter; **Isaf**, ischium articular facet; **Il**, illium; **Isaf**, ischium articular facet; **lc**, lateral condyle; **mae**, ambiens muscle eminence; **mCFLs**, scars for attachment of muscle caudifemoralis longus; **of**, obturator foramen; **pf**, pubic foot; **pv**, posteroventral process; **rP**, right pubis; **sac**, supraacetabular crest; **tc**, tibial condyle; **ts**, trochanteric shelf. Scale bar equals 5 cm.

A fragment of the left ilium comprises the distal portion of the pubic peduncle (Figure 7D). It is fused with the proximal end of the pubis, and preserved in articulation with the left femur (although the latter is dorsally rotated from its natural position). Medially it is also fused to a distal fragment of the second sacral rib. The pubic peduncle is stout, anteroventrally directed and forms the anterior border of a wide perforate acetabulum as in Herrerasaurus (Novas 1994). The supra-acetabular crest forms the straight lateral edge of the pubic peduncle, extending posterodorsally over the acetabulum as in Herrerasaurus.

The pubis is relatively short, its proximodistal length comprising only 63% the length of the femur (Figure 7A–C). By contrast, in Herrerasaurus and Staurikosaurus, the length of the pubis equals 91% (PVL 2566) and 70% the length of the femur, respectively. The two proximal articular surfaces are set at an angle of 130°. The first faces posteriorly and articulates with the ischium whereas the second faces posterodorsally and includes an acetabular section and an articular facet for the ilium. A marked prominence, more distinct than that seen in Herrerasaurus (Novas 1994), is located on the anterolateral margin of the pubis near the iliac peduncle and is presumed to represent the insertion site for the ambiens muscle. The oval obturator foramen is large, its anteroposterior diameter measuring 34% of the anteroposterior width of the proximal end. In Sanjuansaurus the pubis shaft lacks the strong proximal curvature characteristic of Herrerasaurus (Novas 1994), although the lateral margin of the shaft has a similar sinuous curvature in anterior view. The distal portion of the pubis expands and is turned posteriorly to form a pubic “foot” as in Herrerasaurus and Staurikosaurus (Novas 1994; Bittencourt and Kellner 2009). The anteroposterior width of the pubic “foot” is 40% of pubic length, which is slightly less than in Herrerasaurus (43% and 48% in small and large individuals, respectively) (Novas 1994), but greater than in Staurikosaurus (26%).

Both femora are poorly preserved (Figure 7D–H). The left femur is complete and articulated with the ilium, although rotated dorsally from its natural articulation (Figure 7D). Only the proximal and distal ends of the right femur are preserved (Figure 7E). The femur is sigmoid in lateral and anterior views (Figure 7D, E). The anteromedially projecting head lies at an angle of approximately 65° to the transverse axis of the distal end. This is slightly more divergent than in Herrerasaurus (55°; Novas 1994). The size of the head is smaller and narrower transversally than in small individuals of Herrerasaurus (PVSJ 373) that are smaller in overall size than Sanjuansaurus. In proximal view the head is kidney-shaped (Figure 7F), and its proximal surface is smoothly convex as in Herrerasaurus (Novas 1994), although Sanjuansaurus lacks the facies articularis antitrochanterica present in the latter (Novas 1994). The anterior surface of the femoral neck lacks the pronounced anterior trochanter present in Herrerasaurus (Novas 1994). Although partially obscured by deformation and adhering hematite, the trochanteric shelf is present on the lateral surface of the femur (Figure 7E). The shaft of the left femur appears to be more robust than that of Herrerasaurus, although this may be an artifact of preservation. Anteriorly, it has a pronounced keel that extends from the level of the trochanteric shelf proximally to the distal quarter of the femur. The fourth trochanter is semi-elliptical in lateral view and located on the proximal third of the femur (Figure 7E), similar to the condition in Chindesaurus (Long and Murry 1995: Fig. 184). It is longer (one fourth of femoral length), thinner, and seemingly more symmetrical than in Herrerasaurus. A very large, pronounced and rugose protuberance is present on the medial surface of the femur at the level of the fourth trochanter, presumably for the insertion of M. caudifemoralis longus (Figure 7G). In Herrerasaurus this protuberance is relatively small and smooth. The distal end of the femur is expanded. The anterior surface is convex transversally, lacks an intercondylar groove, and has a large attachment scar that extends laterally as in Herrerasaurus (Novas 1994). The posterior surface has a deeper popliteal fossa than in Herrerasaurus (Novas 1994). The crista tibiofibularis is separated by a sulcus from the fibular condyle and projects farther posteriorly than the tibial condyle (Figure 7H). The articular surface of the distal end has a concavity extending from the popliteal fossa medially to the groove between the crista tibiofibularis and the fibular condyle as in Herrerasaurus.

The left tibia of Sanjuansaurus is complete and well preserved (Figure 7I–N). The right tibia is preserved in articulation with the fibula. The distal ends of these bones as well as the right astragalus and calcaneum exhibit some features that appear to be abnormalities rather than artifacts of postmortem compression or crushing. The tibia is slightly shorter than the femur. The tibiofemoral ratio is 0.89, which lies within the range recorded for Herrerasaurus (0.87–0.91) (Novas 1994). The proximal end of the tibia is subtriangular with its long axis directed anteroposteriorly. The cnemial crest projects anteriorly and extends along the proximal one fifth of the tibia. The lateral condyle is posteriorly located as in Herrerasaurus. In cross-section, the proximal half of the shaft is elliptical and the distal half subcircular. In lateral view, the anterior margin of the tibial shaft ventral to the cnemial crest is straight, whereas it is concave in Herrerasaurus (Novas 1994: Fig. 8B). In distal view the distal end of the tibia is subcircular, rather than quadrangular, more closely resembling the condition in Staurikosaurus (Galton 1977) than in Herrerasaurus. The posteroventral process is transversally narrower and dorsoventrally shorter than in Herrerasaurus. The distal end of the right tibia, which as mentioned above appears to be pathologic, has an unusual, tab-shaped lateral expansion of the posteroventral process (Figure 7P).

The relatively slender fibula is subequal to the tibia in length and has transversally flattened proximal and distal ends, the former twice the anteroposterior width at the mid shaft. The shaft is slightly bowed anterolaterally, and has a subtriangular cross-section at mid shaft. Poor surface preservation and breakage of the distal end obscure further details.

**Figure 8.**
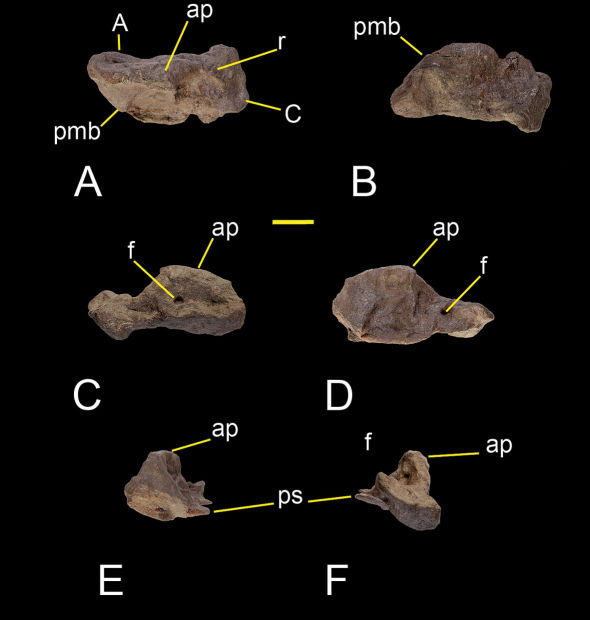
Right astragalus and calcaneum of Sanjuansaurus gordilloi (PVSJ 605). Right astragalus and calcaneum in proximal (**A**), distal (**B**), anterior (**C**), posterior (**D**), lateral (**E**), and medial views (**F**). *Abbreviations*: **A**, astragalus; **ap**, ascending process; **C**, calcaneum; **f**, foramen; **pmb**, posteromedial border; **ps**, posterior shelf; **r**, ridge. Scale bar equals 2 cm.

The astragalus and calcaneum of Sanjuansaurus are fused as in some dinosauromophs (Dromomeron romeri; Irmis et al. 2007), Lagerpeton chanarensis (Romer 1971), heterodontosaurids (Santa Luca 1980), and coelophysoid theropods (Raath 1969). Unlike in many coelophysoids, however, there is no fusion between the crus and proximal tarsals. Whereas the complete fusion of proximal tarsals in Sanjuansaurus might be natural, the distal end of the tibia just above the preserved proximal tarsals appears to be pathologic. Thus we are uncertain whether the observed fusion of the preserved right proximal tarsals is natural or a pathological condition. The astragalus is subtriangular in dorsal view, with a rounded posteromedial border, instead of the distinct posteromedial corner present in Herrerasaurus (Figure 8A, B). The ascending process is tabular, extending transversally from the medial border of the astragalus mediolaterally along the entire width of the astragalus (Figure 8A, B). In Sanjuansaurus, the tip of the ascending process is located at one fifth the length of the lateral border, as in Herrerasaurus, but is close to the anterior border in the former, whereas in the latter it is close to the posterior border. Lateral to its tip, the ascending process continues as a ridge on the proximomedial surface of the calcaneum (Figure 8A). The anterior surface of the ascending process is pierced by a large foramen near its base, as in Herrerasaurus (Novas 1994). The posterior portion of the astragalus is flat with a sharp posterior edge that forms a posteriorly projecting shelf (Figure 8E, F).

A complete left second metatarsal is the only pedal bone preserved. It is straight in dorsal view (Figure 7O). The proximal end is transversely flattened with the long axis directed anterolaterally as in Herrerasaurus (Novas 1994). A pair of distinct articular surfaces for the first and third metatarsals is present on the medial and lateral sides of the proximal part of the shaft. The proximal articular surface is subrectangular in proximal view. The narrow shaft is twisted so that, in distal view, proximal and distal ends have undergone a clockwise rotation of 45°. The distal end of the metatarsal is asymmetrical, with the lateral condyle extending further distally than the medial condyle. The lateral condyle is more developed than the medial and has a deeper collateral ligament fossa. The distal end has a transversely broad dorsal extensor depression to accommodate the base of the proximal pedal phalanx. The depression is bounded proximally by a ridge, which is more prominent laterally than medially. These features also characterize the second metatarsal of Herrerasaurus (Novas 1994).

**Figure 9.**
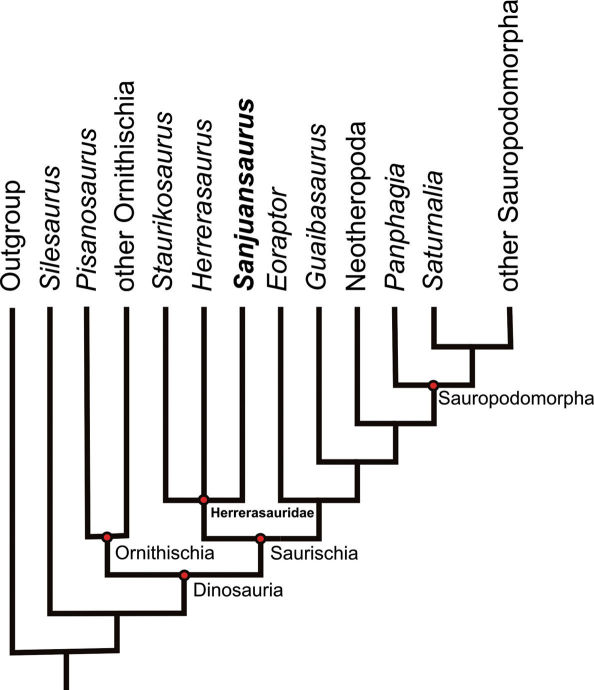
Single most parsimonious tree (MPT) resulting from the present parsimony analysis (tree length 190 steps; consistency index 0.553, retention index 0.593).

## Discussion

Sanjuansaurus gordilloi exhibits several features that allow its distinction from all other known basal dinosaurs:

1.	Shelf-like, posterolaterally directed transverse processes in posterior cervical vertebrae. In Sanjuansaurus from the fifth cervical vertebra back, the transverse processes increase in anteroposterior width and transverse length, and project posteroventrolaterally (Figure 5B), an unusual shape among basal dinosaurs. In other basal dinosaurs the transverse process are shorter or triangular (e.g,, Herrerasaurus, Staurikosaurus, Eoraptor, Tawa, Adeopapposaurus; Sereno et al. 1993; Sereno and Novas 1994; Bittencourt and Kellner 2009; Martinez 2009; Nesbitt et al. 2009).

2.	Neural spines of the sixth to eighth dorsal vertebrae, at least, bearing acute anterior and posterior processes. Sanjuansaurus has distinctive pointed processes extending anteriorly and posteriorly from the apex of the preserved anterior dorsal spines (Figure 5C, D). This feature is unique among known dinosaurs.

3.	Coracoid with long posteroventral process. In Sanjuansaurus the hook-shaped posteroventral process of the coracoid is long and pointed (Figure 6B), similar to that in ornithomimids such as Gallimimus (Osmólska and Barsbold 1990: Fig. 4). In other basal dinosaurs the posteroventral process of the coracoid is less developed (e.g,, Herrerasaurus, Guaibasaurus, Eoraptor, Tawa, Saturnalia; Sereno et al. 1993; Sereno and Novas 1994; Bonaparte et al. 2007; Langer et al. 2007; Nesbitt et al. 2009)

4.	Everted lateral margins of the glenoid. In Sanjuansaurus the rim of the glenoid protrudes laterally in a conspicuous manner (Figure 6A). In the glenoid portion of the bones the margins represent 50% of the thickness of the scapula and the 60% the thickness of the coracoid. In other basal dinosaurs the glenoid portions of the scapula and coracoid are the thicker portions of the respective bones, but they lack the everted lateral margins (e.g, Herrerasaurus, Eoraptor, Panphagia, Tawa, Guaibasaurus, Saturnalia; Sereno et al. 1993;Sereno and Novas 1994; Bonaparte et al. 2007; Martinez and Alcober 2009; Nesbitt et al. 2009)

5.	Short pubis (63% of the femoral length). In Sanjuansaurus the pubis is very short (63% of the femoral length), shorter than that present in other basal surischians (e.g, Herrerasaurus (91%), Staurikosaurus (70%), Tawa (90%), Eoraptor (80%).

6.	Pronounced rugose scar on the medial surface of the femur at the level of the fourth trochanter. In Sanjuansaurus the scar presumably for the insertion of M. caudifemoralis longus is very large, pronounced and rugose protuberance (Figure 7G). In other basal dinosaurs this scar is relatively small and smooth (e.g., Herrerasaurus, Eoraptor, Panphagia, Tawa, Guaibasaurus, Saturnalia; Sereno et al. 1993; Sereno and Novas 1994; Bonaparte et al. 1999; Langer 2003; Martinez and Alcober 2009; Nesbitt et al. 2009)

These autapomorphies allow us to distinguish Sanjuansaurus gordilloi from other known basal dinosaurs. Furthermore, although Sanjuansaurus and Herrerasaurus are similar, the new taxon can be further distinguished from the latter form by three other characters: (1)The scapular blade of Sanjuansaurus is straplike in lateral view (Figure 6A, B), similar in shape than that present in Herrerasaurus, but it is narrower in Sanjuansaurus. The minimum width of the blade (near the base) is 54% the width of the acromial margin, less than that found in either small (83%; PVSJ 407) or very large individuals (60%; PVSJ 053) of Herrerasaurus. (2) The pubis shaft of Sanjuansaurus lacks the strong proximal curvature characteristic of Herrerasaurus (Novas 1994). (3) In Sanjuansaurus the obturator foramen of the pubis is larger. The anteroposterior diameter of the obturator foramen of Sanjuansaurus measures 34% of the anteroposterior width of the proximal end of the pubis, whereas that value is 15% in Herrerasaurus.

### Phylogenetic Position

Phylogenetic analysis resulted in a single most parsimonious tree of 190 steps (consistency index 0.553, retention index 0.593). An implicit enumeration search (Goloboff et al. 2003) and jackknifing (probability of character removal 0.36, 1,000 resampled matrices) were also performed. The topology of the most parsimonious tree is similar to the consensus tree recovered by Martinez and Alcober (2009), differing mainly in resolved positions for Silesaurus and Guaibasaurus. In the present analysis Silesaurus was positioned outside Dinosauria, and Guaibasaurus was positioned as a non-eusaurischian saurischian as in the analysis by Langer and Benton (2006).

The analysis supports the hypotheses that Dinosauria and Herrerasauridae (Staurikosaurus pricei + Herrerasaurus ischigualastensis + Sanjuansaurus gordilloi) are monophyletic and that Herrerasauridae is positioned at the base of Saurischia outside of Eusaurischia, a result similar to that presented by Langer and Benton (Langer and Benton 2006). Within Herrerasauridae, a polytomy was obtained between Staurikosaurus, Sanjuansaurus and Herrerasaurus.

Seven synapomorphies support the clade Herrerasauridae in the consensus tree (characters 20.1, 39.1, 45.1, 46.1, 47.1, 69.2, and 77.1). Only four of those (characters 39, 45, 46, and 77) can be scored in all herrerasaurids, and character 20 is the only one that cannot be determined in Sanjuansaurus. The unambiguous synapomorphies that unite the herrerasaurids in this analysis are the same as those previously identified (Langer and Benton 2006), and do not modify our understanding of the monophyly of Herrerasauridae, although they clearly recover Sanjuansaurus as herrerasaurid.

Other features that support a grouping Sanjuansaurus + Herrerasaurus, but are ambiguous at present include: a narrow “U” shaped antorbital fossa with a promaxillary fenestra located on the anterodorsal border; centrum of the sixth cervical vertebra longest in the cervical series; spine tables on the distal end of the last dorsal and the sacral neural spines; two sacral vertebrae with the addition of one incipient dorsosacral; strap-shaped scapular blade that forms an angle of more than 90° with the acromion; size and shape of the pubic foot; and sinuous lateral border of the pubis in anterior view. There are a few ambiguous character-states shared by Sanjuansaurus and Staurikosaurus but not with Herrerasaurus: shortness of the pubis relative to the femur and the subcircular distal end of the tibia in distal view. The latter character was originally considered as an autapomorphy of Staurikosaurus (Bittencourt and Kellner 2009).

On the other hand, many characters of Sanjuansaurus are unlike those inother herrerasaurids (where known): long band-shaped transverse processes of the distal cervicals; deep lateral and ventral concavity in cervical and dorsal centra; short pubis with wide subcircular obturator foramen; and fused astragalus and calcaneum with a tabular ascending process (if this character-state is not pathological in origin).

### Faunal Considerations

The co-occurrence in the basal portion of the Ischigualasto Formation, of two herrerasaurids (Sanjuansaurus, Herrerasaurus), a basal saurischian (Eoraptor), and two sauropodomorphs (Panphagia, Chromogisaurus) suggests that saurischian dinosaurs were already highly diversified in southwestern Pangea early in the Late Triassic. The new herrerasaurid also represents another large-bodied predatory dinosaur in the Carnian-age Ischigualasto fauna, contrasting with the rarity of carnivorous dinosaurs in the successive Norian assemblage of the overlaying Los Colorados Formation. In that richly represented assemblage, only a single specimen of a coelophysoid theropod has been recovered to date (Arcucci and Coria 2003). Carnivorous crurotarsan archosaurs dominate this Norian fauna, underscoring a complex pattern of faunal change. The faunal assemblages of Ischigualasto Basin strengthen the theory of a complex early radiation of dinosaurs (Brusatte et al. 2010), controlled by a succession of events developed during the Late Triassic, in opposition to models of gradual dominance by competition (Bonaparte 1982; Charig 1984), rapid diversification in the late Carnian (Padian and May 1993); opportunistic radiation in the Norian and Early Jurassic (Benton 1993; Benton 2006), dinosaurian dominance in the Norian (Novas 1996), or early diversification in the Carnian and increase in diversity and abundance in the Norian (Langer et al. 2010).

## Supplementary Material

XML Treatment for Sanjuansaurus
